# Artificial Intelligence in Diagnosis of Long QT Syndrome: A Review of Current State, Challenges, and Future Perspectives

**DOI:** 10.1016/j.mcpdig.2023.11.003

**Published:** 2023-12-18

**Authors:** Negar Raissi Dehkordi, Nastaran Raissi Dehkordi, Kimia Karimi Toudeshki, Mohammad Hadi Farjoo

**Affiliations:** a Department of Pharmacology, Shahid Beheshti University of Medical Sciences, Tehran, Iran; b Department of Cardiology, Shohadaye Tajrish Hospital, Shahid Beheshti University of Medical Sciences, Tehran, Iran

## Abstract

Long QT syndrome (LQTS) is a potentially life-threatening cardiac repolarization disorder characterized by an increased risk of fatal arrhythmias. Accurate and timely diagnosis is essential for risk stratification and appropriate management. However, traditional diagnostic approaches have limitations, necessitating more objective and efficient tools. Artificial intelligence (AI) offers promising solutions by enhancing the accuracy and efficiency of electrocardiography (ECG) interpretation. The AI algorithms can process ECG data more rapidly than human experts, providing real-time analysis and prompt identification of individuals at risk, and reducing interobserver variability. By analyzing large volumes of ECG data, AI algorithms can extract meaningful features that may not be apparent to the human eye. Advancements in AI-driven corrected QT interval monitoring using mobile ECG devices, such as smartwatches, offer a valuable and convenient tool for identifying individuals at risk of LQTS-related complications, which is particularly applicable during pandemic conditions, such as COVID-19. Integration of AI into clinical practice poses a number of challenges. Bias in data gathering and patient privacy concerns are important considerations that must be addressed. Safeguarding patient privacy and ensuring data protection are crucial for maintaining trust in AI-driven systems. In addition, the interpretability of AI algorithms is a concern because understanding the decision-making process is essential for clinicians to trust and confidently use these tools. Future perspectives in this field may involve the integration of AI into diagnostic protocols, through genetic subtype classifications on the basis of ECG data. Moreover, explainable AI techniques aim to uncover ECG features associated with LQTS diagnosis, suggesting new insights into LQTS pathophysiology.

**Article Highlights**

• AI enhances Long QT syndrome diagnosis by improving accuracy.
• Neural networks can extract meaningful hidden patterns associated with Long QT syndrome.
• AI-enabled mobile echocardiography devices provide convenient and accurate QTc monitoring.
• Challenges include bias, patient privacy concerns, and interpretability.
• AI-driven tools can be integrated into diagnostic protocols.

Long QT syndrome (LQTS) is a potentially life-threatening cardiac repolarization disorder characterized by delayed ventricular repolarization, leading to a prolonged QT interval (99th percentile corrected QT interval [QTc] values of ≥470 ms for males and 480 ms for females) on the ECG.^1^^,^^2^ The LQTS may be congenital or acquired, with an estimated prevalence of 1 in 2000 worldwide, and is associated with an increased risk of sudden cardiac death because of life-threatening arrhythmias, such as torsades de pointes (TdP).^3^^,^^4^ Patients with symptomatic LQTS face a considerable 1-year mortality rate of 21%, a number that can be substantially reduced to ∼1% over a 15-year follow-up period with appropriate management.^5^ QT prolongation and TdP are among the significant adverse effects associated with a variety of cardiac and noncardiac medications, and in some cases can lead to the withdrawal of certain medications from the market.^6^ Given its ominous consequences, early and accurate diagnosis of LQTS is critical for appropriate risk stratification and the implementation of effective management strategies.

The current diagnosis of LQTS typically involves assessment of clinical presentation, family history, interpretation of ECG findings, and in some cases, genetic testing.^7^ However, only 50% of patients with LQTS-related mutations manifest symptoms, and ∼25% may present with a normal QTc on the ECG.^8^ Inconclusive clinical manifestations and ECG findings can lead to diagnostic errors and may delay appropriate management. Therefore, there is a growing need for more objective and efficient diagnostic tools that help in the detection and management of LQTS.

In recent years, there has been a growing interest in the application of artificial intelligence (AI) and machine learning (ML) in the field. Artificial intelligence algorithms can effectively process large volumes of ECG data, extract meaningful patterns and features, and make predictions on the basis of the learned patterns.^9^ And ML enables AI systems to continuously learn from new data and adapt their algorithms to improve performance over time,^10^ enhancing diagnostic accuracy.^11^

In this review article, we aim to explore the application of AI in the diagnosis of LQTS from ECG. We summarize the capabilities of AI in recognizing specific ECG patterns associated with LQTS, evaluate their diagnostic accuracy compared with traditional methods, and discuss their potential clinical impact. In addition, we provide an overview of the challenges and future directions of AI in LQTS.

### Artificial Intelligence, Machine Learning and Neural Networks for Data Analysis

In today's rapidly advancing technological landscape, AI has emerged as a game-changer, revolutionizing the way we interact with computers and machines. Artificial intelligence is defined as a system that can effectively understand and interpret data from external sources, gain insights from that data, and use the acquired knowledge to accomplish specific objectives and tasks by adapting and adjusting as needed (Figure).^9^

**Figure:**
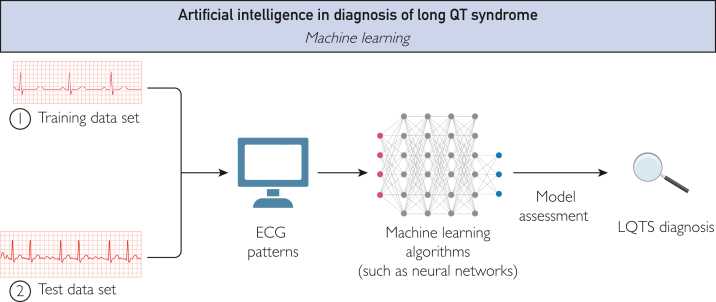
Artificial intelligence can be used in cardiology to effectively understand and interpret data from ECG data, gain insights from that data, and use the acquired knowledge for diagnostic purposes.

The ML techniques, which are a subset of AI, can be categorized into 4 types: supervised, unsupervised, semi-supervised, and reinforcement. Supervised learning, the main type of ML used in LQTS interpretation, involves using labeled data for tasks like classification,^12^ and encompasses a wide range of algorithms, such as neural networks.^13^ Neural networks are a type of supervised ML algorithm inspired by the structure and function of the human brain. They consist of interconnected nodes (artificial neurons) organized in layers. Through a process known as training, the neural networks learn to process and transmit information through interconnected nodes. Each neuron takes inputs, performs computations, and produces an output that is passed on to higher level neurons^14^ allowing them to learn from data, recognize patterns, and make predictions.^15^ Neural networks allow advancements in areas such as image and speech recognition, natural language processing, and autonomous decision-making.^16^ These properties are used for training neural networks by large datasets of annotated ECGs to recognize specific ECG features associated with certain heart conditions, such as LQTS, enabling automated and reliable diagnosis.^17^

Multi-layered neural networks can extract features by themselves, using a method called deep learning (DL), a subtype of ML. Various types of neural networks are used for DL on the basis of their connection and calculation methods. They include recurrent neural networks, convolutional neural networks, and deep neural networks.^18^ Among them, the convolutional neural networks stand out as the most commonly employed algorithm because of their ability to automatically identify relevant features without the need for human supervision. Their versatility and effectiveness make them a popular choice for various applications in DL such as ECG interpretation.^18^

### AI in LQTS Diagnosis

The clinical importance of applying AI and specifically neural networks in the interpretation of ECGs lies in their ability to overcome some of the limitations of traditional diagnostic approaches. First, AI algorithms can process ECG data rapidly, allowing for real-time analysis and prompt identification of individuals at a high risk of sudden cardiac death.^19^ This can be particularly valuable in emergency settings, where immediate decision-making is crucial. Second, AI-based systems can provide consistent and standardized interpretations of ECGs, reducing interobserver variability and improving diagnostic accuracy.^20^ By minimizing the risk of misdiagnosis, these systems have the potential to facilitate early intervention and prevent fatal arrhythmic events. Furthermore, AI algorithms can analyze large volumes of ECG data and extract meaningful features that may not be readily apparent to the human eye.^21^ This can aid in the identification of subtle abnormalities, provide deeper insights into the pathophysiology of cardiac arrhythmias, and potentially uncover novel ECG findings associated with disease severity and prognosis.

The diagnosis of LQTS is on the basis of the LQTS diagnostic criteria score, also known as the Schwartz-score, which considers ECG findings, clinical history, and family history.^22^ A Schwartz-score of ≥3.5 indicates a high specificity (99%).^23^ Genetic testing is recommended if the Schwartz-score is ≥3, to help identify disease-causing mutations, provide a definitive diagnosis, and identify at-risk family members.^24^ Although LQTS can be easily diagnosed in clear-cut cases, patients presenting with indefinite diagnostic criteria pose several challenges. In these cases, the resting ECG may not show abnormalities (12%-30% of cases), patients may not exhibit any symptoms, and family history may not provide conclusive evidence.^25^ In addition, there can be variations in measuring and interpreting the QT interval on the ECG; the determination of the upper limit of normal QT interval duration is complex, and heart rate corrections can be unreliable.^26^ Therefore, the development of more objective and efficient diagnostic tools would be beneficial in improving LQTS diagnosis.

Artificial intelligence-driven analysis of ECG data has shown promising results for the detection of LQTS. By using ML algorithms, AI models can analyze ECG waveforms and extract key features indicative of LQTS, such as QT interval duration and T-wave morphology. The Table provides a summary of different studies that used AI related to the interpretation of ECGs and the diagnosis of LQTS.

**Table:** A Summary of Different Studies that Utilized AI Algorithms and Techniques for Interpretation of ECGs and the Diagnosis of LQTS

Reference Year	Objective	Dataset (Study Population)	Accuracy	Sensitivity	Specificity	AUC	F1	Precision	Recall
Prifti et al,^27^ 2021	Assess performance of a CNN for monitoring QTc using ECG data	Generepol, healthy controls receiving Sotalol, patients with cLQTS, patients with diTdP	0.948	-	-	0.98	0.470	0.955	0.927
Bos et al,^28^ 2021	Develop AI-ECG model to distinguish LQTS and classify genetic subtypes	Patients with confirmed LQTS based on positive genetic tests, control group of evaluated patients	82.50%	83.70%	80.60%	0.9	83.60%	-	-
			(78.7% in predicting the genotype status)			(0.863 for concealed QTc)			
Aufiero et al,^29^ 2022	Develop DL models for diagnosis of LQTS using a large dataset of ECGs	Controls, genetically proven LQTS patients	-	84 for LQTS1	96 for LQTS1	0.90 for LQTS1	-	-	-
				90 for LQTS2	95 for LQTS2	0.92 for LQTS2			
				87 for LQTS3	92 for LQTS3	0.89 for LQTS3			
Draelos et al,^30^ 2022	Develop gene-specific ML models for examining pathogenicity of variants in heritable channelopathies	Curated datasets of pathogenic and benign variants	80% for LQTS1	84 for LQTS1	-	0.8739 for LQTS1	-	0.8246 for LQTS1	-
			77% for LQTS2	90 for LQTS2		0.8336 for LQTS2		0.6877 for LQTS2	
			89% for LQTS3	87 for LQTS3		0.7918 for LQTS3		0.2854 for LQTS3	
Doldi et al,^31^ 2022	Compare XceptionTime and FCN models for diagnosing LQTS	Genetically confirmed LQTS patients, control group	FCN: 83.6%	FCN: 82.6%	FCN: 84.7%	FCN: 0.9	FCN: 66%	-	-
			XceptionTime: 91.8%	XceptionTime: 92.9%	XceptionTime: 90.8%	XceptionTime: 0.97	XceptionTime:83.2%		

Abbreviations: AI, artificial intelligence; AUC, area under the curve; CNN, convolutional neural network; cLQTS, congenital long QT syndrome; DL, deep learning; diTdP, drug-induced torsades de pointes; ECG, electrocardiogram; FCN, fully convolutional network; LQTS, long QT syndrome; ML, machine learning; QTc, corrected QT interval

A study aimed to assess the performance of CNN for monitoring QTc using ECG data from 4 distinct cohorts: healthy patients (Generepol), healthy controls receiving sotalol (Pharmacia), patients with congenital long QT syndrome (cLQTS), and patients with drug-induced torsades de pointes (diTdP). To train the CNN, specific alterations in the ECG caused by sotalol were targeted as a representative model for IKr blockade. The IKr blockade is the primary mechanism by which drugs induce QTc prolongation, increasing the risk of TdP. The ECG recordings underwent a review process, including filtering, and the QTc intervals were calculated using Fridericia’s formula. Subsequently, data were standardized to facilitate the training and testing of the AI models. Two types of models were developed: a multilead model (I, II, and V1-6) using 8 independent leads simultaneously, and a unilead model analyzing each lead independently. Both models used a CNN architecture and were trained and tested on the Generepol cohort, then evaluated on the other cohorts. The models provided a score indicating the likelihood of sotalol intake, with lead II performing the best among the single-lead models.

Performance assessment included measurements such as accuracy, recall, precision, F1 score, and area under the curve-receiver operator characteristic. Evaluation was done at both the ECG and patient level, averaging multiple recordings to generate risk score. The CNN models successfully identified likelihood of sotalol intake. Furthermore, iterative dropping of ECG segments and assessing their impact revealed that J-T peak interval features were crucial for distinguishing sotalol intake.^27^

Identifying patients with a normal-looking ECG is crucial because ∼25% of patients with LQTS exhibit concealed LQTS with a normal QTc interval at rest.^22^ The LQTS has multiple subtypes associated with specific gene mutations, with the 3 major subtypes being LQT1, LQT2, and LQT3. These subtypes are primarily caused by mutations in specific ion channel genes: KCNQ1 for LQT1, KCNH2 for LQT2, and SCN5A for LQT3. Collectively, these 3 subtypes account for ∼90% of all genetic cases where a sequence variation has been identified.^3^ In a diagnostic case-control study, researchers retrospectively reviewed patients with a confirmed diagnosis of LQTS on the basis of positive genetic tests and a control group of patients who were evaluated for LQTS but subsequently discharged without a diagnosis.^28^ Demographic characteristic data, clinical history, genetic information, and ECG data were collected for all patients. A CNN model was developed using a portion of the patient data and tested on the remaining patients. The AI-ECG model aimed to distinguish LQTS from a normal QTc interval and classify the genotypic subtypes of the syndrome. The AI model employed unsupervised feature extraction, analyzing the entire ECG waveform across all 12 leads, reporting the ability to identify subtle features in the ECG waveform that may not be apparent to expert cardiologists.

The AI-ECG model found improved accuracy in LQTS detection compared with the QTc measurement alone and found potential for distinguishing LQTS genetic subtypes. In terms of LQTS detection, the AI-ECG model outperformed the QTc alone in distinguishing patients with LQTS from those who were dismissed as normal. In addition, the model also successfully differentiated these 2 populations even when the QTc interval was within the normal range of 450 milliseconds. When evaluating concealed LQTS, the AI-ECG also outperformed the QTc alone, and in terms of LQTS genotype classification, the AI-ECG was able to differentiate the 3 major genetic subtypes. It achieved the highest performance in distinguishing LQT2 from LQT1 and LQT3, in addition to performing well in distinguishing LQT1 from LQT2 or LQT3. The lowest performance was observed when identifying patients with LQT3. In addition, the AI-ECG model was evaluated using a synthetic dataset with different prevalences, and it maintained high performance in terms of AUC, sensitivity, and specificity, whereas the positive predictive value decreased with increasing prevalence. The AI-ECG model achieved a high accuracy in predicting the genotype status of LQTS before undergoing genetic testing, highlighting the potential of AI-driven analysis of ECG data to accurately identify and anticipate LQTS cases.^28^ Although successful in determining genetic subtype classification on the basis of ECG input, we did not find any study elucidating the decision-making process of AI in this regard, and the issue remains a black box. However, it's worth noting that with the emergence of new technologies, particularly in the field of explainable AI, there is hope for a more transparent understanding of AI decision-making processes. This could potentially unveil critical associations between specific segments of the ECG and distinct mutations within specific genes, ultimately shedding light on the comprehensive classification of genetic subtypes. Such advancements may pave the way for a deeper comprehension of the intricate relationship between ECG data and underlying genetic mutations, unlocking new doors for diagnosis of LQTS.

Another study aimed to develop DL models for diagnosis of LQTS by using a large dataset of 12-lead ECGs consisting of 10,000 controls and 458 genetically proven LQTS patients (172 LQTS1, 214 LQTS2, and 71 LQTS3 patients). The DL models outperformed automatic and manual QTc measurement in identifying LQTS patients. In a performance comparison with an international expert in congenital LQTS, the DL models outperformed the cardiologist in terms of specificity but had similar sensitivity.^29^

To examine the pathogenicity of variants with uncertain clinical significance in the genes related to heritable channelopathies such as LQTS, the researchers developed gene-specific ML models.^30^ The models were trained using curated datasets of pathogenic and benign variants and were validated using cross-validation techniques. The study successfully validated the accuracy of ML models in predicting pathogenicity and confirmed the reliability of ML predictions. Furthermore, when retrospectively applied to channelopathy cases, the ML-based predictions exhibited a high level of accuracy in predicting the clinical phenotype across KCNQ1 and KCNH2 variants, emphasizing the potential clinical use of ML models.^30^

Another retrospective study compared traditional CNN models, such as fully convolutional neural networks (FCN) with modern CNN models such as XceptionTime to evaluate the enhanced predictive accuracy. The XceptionTime model, originally designed for multichannel surface electromyography signals, was adapted for 12-lead ECG analysis. The researchers used genetically confirmed LQTS patients (n=124, such as 41 concealed cases) and a control group consisting of 161 patients without LQTS or a history of QT-prolonging drugs, but with other cardiac or noncardiac diseases. The XceptionTime model found superior performance compared with the FCN. Both CNN models consistently found high AUC values and optimum ratios between specificity and sensitivity. The XceptionTime model also outperformed the FCN in terms of the F1 score. The results were consistent regardless of age, QTc, and the presence of cardiovascular comorbidities offering an alternative to former CNN models.^31^ The capabilities of ML models extend beyond diagnostic abilities. In terms of QT-monitoring, they enable the implementation of risk stratification strategies that are applicable in clinical practice. By continuously monitoring QT intervals, CNN models can detect changes in the ECG pattern as early as 24 hours before the occurrence of TdP. This advanced detection capability allows for the identification of individuals who may appear normal but are at a higher risk of experiencing potentially lethal LQTS-associated arrhythmias.^32^ Even though their QTc values fall within the normal range, these individuals may still need to avoid medications that could prolong the QT interval and may benefit from prophylactic beta blocker administration. In addition, finding these individuals can help identify at-risk family members.^28^To develop an integrated risk-prediction model for drug-induced QT prolongation, a study applied different ML methods to electronic health record data. A total of 35,639 patients who received medications known to prolong the QT interval and had an ECG within 24 hours after drug administration were analyzed. 4558 patients (12.8%) developed a QTc interval longer than 500 ms, while the remaining 31,081 patients (87.2%) had an interval below 500 ms. The demographic characteristics, including age and gender, were similar between the 2 groups. According to the study findings, the deep neural networks model reported superior classification performance in predicting the development of a long QT interval compared with the other ML methods, with an F1 score of 0.404 and an AUC of 0.71.^6^

### Explainable AI Sheds Light on the Decision-Making Process of Deep Learning Models

Artificial intelligence and especially DL models excel in predictive power by automatically learning complex hierarchical representations from vast amounts of data. However, they lack transparency and interpretability for the human brain. This lack of interpretability poses challenges in trusting and comprehending their decisions by physicians, especially in critical domains like arrhythmias.^33^ Interpretability is crucial for assessing prediction models, as it helps understand the model's recommendations and determine which clinical features were most predictive, helping clinicians construct a meaningful decision support approach and reduces the risk of bias due to data shifts.^34^

The ML models face a trade-off between their performance and explainability. On one hand, there are black-box models like DL, which achieve state-of-the-art performance but are difficult to interpret. On the other hand, there are white-box or glass-box models like linear models that are more explainable but often have lower performance because of their simpler design.^33^^,^^34^

The need for trustworthy, fair, robust, and high-performing models that are also understandable in real-world applications has led to the resurgence of the explainable AI. Explainable AI focuses on understanding and interpreting the behavior of AI systems, which had previously received less attention as the emphasis was primarily on predictive power.^33^

Although human evaluation relies primarily on QTc interval information, AI input consist of the entirety of the ECG.^28^ Given the marked diversity in input data containing complex electrical signals in the ECG, compared with a single QTc number, and considering the extensive training on vast amounts of data compared with the limited number of ECGs used by humans, it is not surprising that AI exhibits a heightened capacity for distinguishing LQTS patients from healthy controls. Artificial intelligence exhibits superior diagnostic performance compared with expert clinicians, particularly excelling in identifying cases of concealed LQTS, which may appear normal to clinicians. This suggests that AI leverages a broader range of ECG findings beyond the QT interval alone.

A study aimed to determine the value of supervised learning assessment of T-wave morphology markers obtained from 12-lead ECGs in identifying LQTS patients compared with standard clinical parameters. The study population consisted of LQTS patients with confirmed pathogenic variants in KCNQ1, KCNH2, or SCN5A genes, and genotype-negative family members who served as healthy controls. In this study, various T-wave morphology features, encompassing area, biphasic pattern, amplitude, skewness, kurtosis, and asymmetry across leads VR, VL, VF, V1-V6, were extracted from 12-lead ECGs. These features were then automatically computed using a tailored software. Machine learning classification models were subsequently trained and evaluated using these features.^35^ Previous studies have established the pivotal role of T-wave morphology in the diagnosis of LQTS, aligning with its pathophysiology.^36^ Two models were compared. The baseline model incorporated age, QT interval, and QTc-intervals as inputs, and the extended model integrated T-wave morphology features in addition to the baseline parameters. The extended model reported improved AUC scores, in addition to a major reduction in misclassified LQTS patients compared with using QTc scores. The study reported the potential applicability of T-wave morphology features obtained from standard 1 ECGs in improving the diagnosis of LQTS, providing additional value beyond traditional clinical parameters.^35^

In another study, an explainable AI technique was applied to understand a DL model’s decision-making process and localized the most important region of ECG used by the model for classification. Interestingly, the explainable AI technique identified the onset of the QRS complex as the most relevant region.^29^ This finding challenges current concepts of LQTS pathophysiology, which focus on the repolarization phase of the action potential and T-wave abnormalities in LQTS and suggests alterations in the initial ventricular depolarization in carriers of disease-causing LQTS mutations. The potential impact of ventricular depolarization, as indicated by the QRS changes identified by the AI model, introduces new possibilities regarding LQTS pathophysiology and suggests involvement of ion channels other than, or in addition to the potassium channels. Theoretically, this finding could lead to improved understanding of the underlying mechanisms responsible for LQTS and provide new treatments.

As 12-lead ECGs are commonly employed in training AI models, discerning the relative importance of individual leads remains challenging. However, a number of studies conducted on single-lead mobile ECG (mECG) devices have yielded results comparable with those of 12-lead studies in diagnosis of LQTS, implying that single-lead information could potentially lead to similar results for AI in diagnosing LQTS.

### AI-Driven QTc Monitoring with mobile ECG Devices

The advancements in AI, along with the availability of mECG devices such as smartwatches and activity trackers, have opened up promising opportunities for assessing and monitoring QTc prolongation in a widespread manner, akin to measuring vital signs. These AI-enabled mECG devices offer health care professionals a valuable and straightforward tool to remotely identify and monitor individuals at risk of congenital and acquired LQTS-related morbidity and mortality. The evaluation of QTc using these devices effectively separates it from the conventional 12-lead ECG, providing a convenient approach for QTc assessment.^37^ These AI tools have reported consistent diagnostic accuracy, regardless of whether a 1-lead or 12-lead ECG recording is used. These features are instrumental in developing a user-friendly remote monitoring system, which can be operated by patients themselves, to detect potentially hazardous ECG changes and implement timely interventions to mitigate the risk of arrhythmias. This innovative risk-monitoring system not only surpasses existing methods but also has the potential to transform the way we approach the use of QT-prolonging drugs. By replacing the current strategy of pre-prescription risk prediction, in conjunction with real-time monitoring facilitated by this accurate system, we can minimize adverse arrhythmic events and increase confidence in prescribing these drugs. Consequently, this system has the potential to address physician concerns and reduce unnecessary caution in prescribing QT-prolonging drugs for diverse patient populations with various medical conditions.^32^

The COVID-19 pandemic has further emphasized the need for alternative long distance monitoring methods to minimize risks and conserve resources. Traditional QTc monitoring, relying on 12-lead ECG systems and trained technicians, is limited to specific clinical settings. In this context, the integration of AI and mECG devices empowers patients to conduct QTc measurements by themselves, in the comfort of their homes, offering opportunities for early identification of at-risk individuals and potentially life-saving interventions.^37^

A prospective observational study was conducted to compare QTc measurements by using an AI algorithm on smartwatch single-lead ECGs (SW-ECG) with those derived from conventional 12-lead ECGs in early-stage COVID-19 patients treated with the hydroxychloroquine-azithromycin regimen. The results found good agreement between the 2 methods. The mean QTc duration was 407±26 ms on the 12-lead ECG vs 407±22 ms on SW-ECG, with a negligible bias of −1 ms. In 98.2% of patients, the difference between the measures was less than 50 ms.^38^

In another study, using the FDA-approved AliveCor mECG device, an AI DL model was trained on over 1.6 million ECGs, and achieved accurate detection of QTc prolongation, particularly a QTc of ≥500 ms.^37^ Furthermore, even by lowering the threshold to ≥460 ms, which represents the 99.9th percentile QTc value for healthy individuals, the AI model still exhibited comparable performance in detecting QTc prolongation. With further improvements, AI-assisted single-lead ECGs could become a viable option for QTc interval monitoring in epidemic conditions.

### Challenges

The potential impact of AI on LQTS diagnosis is substantial. Integration of AI models into routine clinical practice offers the ability to enhance the accuracy of LQTS diagnosis and subtype classification. Despite the promising results of AI-ECG models, it is imperative to address limitations before the widespread clinical implementation of DL models.

The potential bias in data gathering and the information source used to train ML algorithms is a major concern. Currently, these models have primarily been developed and tested on specific cohorts or single centers, such as specialized LQTS centers. Consequently, their applicability to more diverse populations remains uncertain, necessitating further investigation for their globalization. Assessing the performance, reliability, and accuracy of these models across diverse health care settings is crucial to ascertain their robustness and validity in real-world scenarios. Further research, including large-scale population-based studies and expanded screening for other clinical disorders, is warranted before formal recommendations are made by medical societies.^37^

Another critical aspect before embracing AI in LQTS diagnosis and management is addressing challenges related to interpretability, understanding, and trust in prediction algorithms. Achieving transparency and providing clear explanations for the predictions made by these models are essential to instill confidence into health care professionals and patients alike. Developing guidelines and standards for interpreting AI-ECG model outputs is paramount to ensure their effective integration into clinical practice, thereby enhancing patient care while minimizing potential risks. By establishing these frameworks, health care professionals can better comprehend the intricacies of AI-driven predictions, fostering a greater trust in these models.

In addition, patient privacy emerges as a prominent concern when using large datasets, electronic health records, and mobile ECG devices. The utilization of such data sources poses the risk of breaching patient privacy and the leakage of personal information without patients being adequately informed about privacy risks. As the implementation of AI-ECG models expands, it is crucial to establish strict data protection measures and informed consent processes to safeguard patient privacy rights and maintain public trust in the health care system. Furthermore, the impact of the widespread application of AI-ECG models in general or primary care populations remains largely unknown.

Another challenge is the potential misclassification of healthy controls as LQTS patients by ML algorithms. Although AI-ECG models have shown promising performance in distinguishing LQTS cases from healthy individuals, there have been instances where false positives were identified, leading to healthy controls being incorrectly classified as LQTS patients.^35^ This can have significant implications, as it may result in unnecessary diagnostic testing, increased health care costs, and potential psychological distress for those individuals.

### Future Directions

The integration of AI in the diagnosis and risk stratification of LQTS holds great promise. The AI models have shown considerable potential in providing genetic subtype classifications based solely on ECG data. In addition, incorporating this initial genetic classification into established risk assessment tools like the Schwartz criteria could significantly enhance risk assessment.^28^ This is particularly crucial in cases of concealed LQTS, where a seemingly normal QT interval might lead to a lower Schwartz-score and potential diagnostic oversight, in addition to preventing the prescription of potentially harmful QT-prolonging drugs and identifying at-risk family members.

As genetic technology continues to advance, an ever-expanding pool of genetic information in inheritable arrhythmias like LQTS is gathered, leading to challenges in the evaluation of rare variants. Although there are guidelines for this purpose,^39^ due to the rarity of LQTS and the limited data available for many of these variants, accurate interpretation of these unusual genetic variants remains a challenge for clinical application.^40^ Notably, there is a paucity of research integrating genetic data into LQTS diagnosis. Further investigation for including rare variant genetic testing data in ML could enhance the efficacy of ML approaches, provided that variant interpretation is performed rigorously and in conjunction with clinical data.^40^

To further improve clinical care, the development of more advanced explainable AI techniques holds great promise, enabling the identification of ECG features previously unknown for LQTS diagnosis. By these techniques, a deeper understanding of LQTS pathophysiology can be achieved, leading to enhanced diagnostic accuracy.

## Potential Competing Interests

All authors declare that there are no conflicts of interest.
